# Laser-induced graphene as a versatile platform for colloidal quantum dot heterostructure photodetectors

**DOI:** 10.1039/d5ra07157g

**Published:** 2025-12-02

**Authors:** Jiyoon Oh, Donggu Lee, Kyoungeun Lee, Yeyun Bae, Jaeyeop Lee, Yeong Uk Kim, Byeong Guk Jeong, Jaehoon Kim, Hanchul Cho, Jeongkyun Roh

**Affiliations:** a Department of Electrical Engineering, Pusan National University Busan 46241 Republic of Korea jkroh@pusan.ac.kr; b Extreme Process Control Group, Korea Institute of Industrial Technology Busan 46938 Republic of Korea hc.cho@kitech.re.kr; c Department of Semiconductor Engineering, Gyeongsang National University Jinju Gyeongnam 52828 Republic of Korea; d Institute for Environment and Energy, School of Chemical Engineering, Pusan National University Busan 46241 Republic of Korea; e Department of Electronic Engineering, Gachon University Seongnam-si, Gyeonggi-do 13120 Republic of Korea

## Abstract

The growing demand for high-performance, flexible, and low-cost photodetectors has driven research interest in heterostructure-based architectures that integrate materials with complementary optical and electronic properties. In this study, we developed a heterostructure photodetector platform based on laser-induced graphene (LIG) and lead sulfide colloidal quantum dots (QDs). LIG, which is directly patterned on polyimide *via* laser irradiation, offers scalable mask-free fabrication with mechanical flexibility and excellent conductivity. To enhance charge transfer between the QDs and LIG, solid-state ligand exchange was performed, replacing the long-chain oleic acid ligands with short iodide ligands. This modification significantly improved charge transfer efficiency, resulting in a high photo-to-dark current ratio (>100), responsivity approximately 20 A W^−1^ with excellent linearity across a wide range of light intensities, and specific detectivity exceeding 10^11^ Jones. Furthermore, the versatility of LIG formation was demonstrated through its compatibility with various polyimide substrate formats, proving its potential for fabricating flexible, large-area, and customizable electrode patterns. These findings highlight the application potential of LIG-QD heterostructures as a platform for next-generation flexible optoelectronic devices.

## Abstract 2

Maskless and simple laser fabrication produces porous laser-induced graphene (LIG) that serves as an excellent platform for integrating PbS quantum dots into high-performance heterostructure photodetectors.

## Graphical abstract

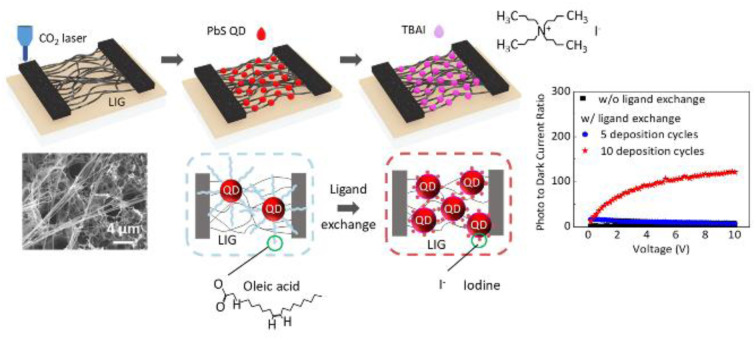

## 1. Introduction

Photodetectors are essential components of modern optoelectronic systems and play a critical role in a wide range of applications including optical communication, environmental monitoring, medical diagnostics, imaging technologies, defense, and industrial automation. The rapid growth of the Internet of Things and wearable electronics has further intensified the demand for high-performance, flexible, lightweight, and cost-effective photodetectors. These devices must satisfy requirements such as high sensitivity, mechanical durability, and compatibility with large-area fabrication while maintaining low production costs. A promising route for achieving these goals is the use of heterostructure-based photodetectors.^1–7^ By integrating two different types of materials, typically one with high optical sensitivity and the other with superior electrical conductivity, heterostructures enable synergistic enhancements in photodetection performance. Such architectures have demonstrated improved sensitivity, a broader spectral range, faster response times, and improved device stability.^8–10^ Various material combinations, such as two-dimensional (2D) materials with organic semiconductors,^11^ 2D materials with perovskites,^12,13^ graphene with perovskites,^14^ and metal oxides with quantum dots (QDs), have been explored.^15–17^ Among these, graphene-QD heterostructures have gained particular attention owing to the exceptional electrical conductivity and mechanical flexibility of graphene, combined with the size-tunable bandgap and high absorption coefficient of QDs.^18–21^

Traditionally, graphene-QD heterostructure photodetectors have been fabricated using chemical vapor deposition (CVD)-grown graphene. Although CVD graphene offers high-quality films with excellent uniformity, its production involves high-temperature processes that require additional lithographic patterning steps, which increase manufacturing costs and limit scalability for industrial applications. Therefore, the development of alternative, efficient, and scalable methods for producing patterned graphene is critical for advancing flexible optoelectronic technologies.

In this study, we propose a laser-induced graphene (LIG)-based platform for constructing graphene-QD heterostructure photodetectors. Unlike conventional CVD-grown graphene, LIG can be patterned directly on polyimide substrates *via* direct photothermal conversion under ambient conditions without lithography or transfer steps, providing a cost-effective and scalable alternative.^22,23^ Moreover, the intrinsic porous morphology of LIG offers an enlarged interfacial contact area with QDs, facilitating efficient charge transfer. We optimized the laser irradiation conditions to form filament-like graphene networks in the channel region, and subsequently integrated PbS QDs with the LIG electrodes. To further enhance device performance, the surface ligands of PbS QDs were engineered *via* solid-state ligand exchange to promote efficient charge transfer. Consequently, the fabricated LIG-QD heterostructure photodetector exhibited high photosensitivity and robust photodetection characteristics. These results demonstrate the potential of LIG-based heterostructures as a low-cost and scalable platform for next-generation optoelectronic devices, and suggest promising opportunities for future development of flexible and broadband photodetectors.

## 2. Experimental

### 2.1. Materials

PI solution (poly(pyromellitic dianhydride-*co*-4,4′-oxydianiline), amic acid solution), PbS (oleic acid coated, fluorescence wavelength of 900 nm, 10 mg mL^−1^ in toluene), and tetrabutylammonium iodide (TBAI) were purchased from Sigma-Aldrich. TBAI was dissolved in methanol at a concentration of 10 mg mL^−1^ prior to use. Methanol (≥99.9%) was obtained from Samchun Chemicals, and glass substrates were sourced from AMG.

### 2.2. Device fabrication and characterization

Photodetectors were fabricated on glass substrates, which were sequentially cleaned in acetone, isopropanol, and deionized water by ultrasonication for 20 min each. After drying, the PI solution was spin coated onto the substrate, followed by thermal curing to form a uniform PI film (45 µm). LIG electrodes were then patterned using a CO_2_ laser (C30, Coryart, Korea) under optimized conditions (scan power: 9 W, speed: 400 mm s^−1^, gap: 0.05 mm). Laser irradiation thermally decomposed PI, forming graphene patterns without the need for additional lithography. Following LIG formation, oleic acid capped PbS QDs (10 mg mL^−1^ in toluene) were spin-coated onto the channel region between LIG electrodes at 2500 rpm for 10 s, followed by a 30-s settling period. A drop of TBAI solution in methanol was then applied to initiate solid-state ligand-exchange, allowing the film to set for 2 min. Subsequently, methanol was spin-coated at 2500 rpm for 30 s to remove the excess ligands and byproducts. This deposition–exchange–rinsing cycle was repeated to obtain the desired thickness for the QD active layer. To protect the device from environmental exposure, a CYTOP (AGC Chemicals Company) encapsulation layer was spin-coated at 3000 rpm for 60 s and thermally annealed at 90 °C for 1 h under a nitrogen atmosphere. The electrical performance was measured using a Keithley 2450 source-measurement unit. The optical response and spectral measurements were performed using a CS-2000 spectroradiometer and a SimuLight SS-LED50S solar simulator (McScience Inc., Suwon, Republic of Korea) under controlled illumination conditions with varying light intensities.

## 3. Results and discussion

**Fig. 1:**
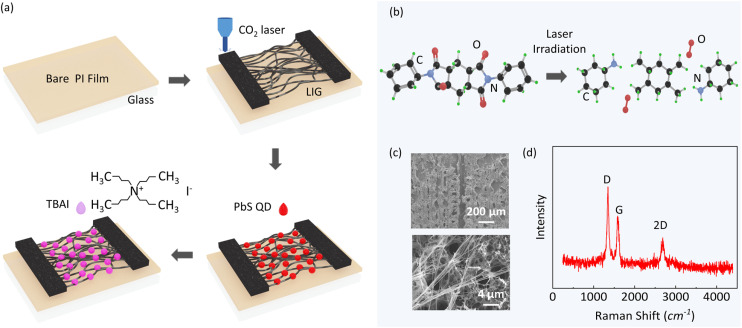
(a) Schematic of the fabrication method for LIG-QD heterostructure photodetectors. (b) Graphene formation mechanism by laser irradiation. (c) SEM images of LIG electrodes showing filament-like network between electrodes. (d) Raman spectra of LIG exhibiting characteristic peaks of graphene.

Compared with CVD-grown graphene, LIG offers a promising alternative for the rapid and cost-effective production of high-surface-area graphene without the need for expensive equipment. Using LIG electrodes, devices can be fabricated directly on flexible substrates with arbitrary patterns, eliminating the need for conventional photolithography. The overall fabrication process for the photodetector is illustrated in Fig. 1a.

First, a PI solution was spin-coated onto pre-cleaned glass substrates to form a uniform PI film (thickness of 45 µm). Subsequently, the desired electrode pattern was formed on the PI film using a CO_2_ laser. After forming the LIG electrodes, the channel region was coated with PbS QDs capped with oleic acid (10 mg mL^−1^ in toluene, emission peak wavelength of 900 nm). To enhance the charge transfer from the QDs to LIG, a ligand exchange was performed using TBAI, replacing the long-chain oleic acid ligands with shorter ones. This exchange improved the electronic coupling between the QDs and LIG, thereby improving the overall device performance. A subsequent spin-coating step using methanol was employed to remove the residual ligands and surface impurities, resulting in a cleaner and more uniform film, which is critical for improving both the device performance and operational stability. The photodetector was fabricated by repeating the deposition process to achieve the desired thickness of QDs.

Fig. 1b illustrates the LIG formation process. When a CO_2_ laser is focused onto a carbon-rich PI film, the localized thermal energy breaks chemical bonds such as C–O, C=O, and C–N, releasing oxygen and nitrogen in gaseous forms. The remaining carbon atoms rearrange to form graphitic or graphene-like structures.^24,25^ The physical and electrical properties of the resulting LIG can be finely tuned by adjusting laser parameters such as power, scanning speed, and focal depth. These parameters directly influence the surface morphology and sheet resistance of LIG.^26^ We optimized laser power and scanning speed by measuring the sheet resistance and morphology of LIG (Fig. S1–S3). As the laser power increases, the ablation and localized vaporization of the polyimide substrate become more pronounced, leading to the formation of a highly porous and interconnected carbon network. However, excessive porosity interrupts the conductive pathways and increases the sheet resistance, despite providing a larger interfacial area for QD loading. Therefore, an optimal balance between porosity and conductivity is essential to ensure both efficient charge transport and strong interfacial coupling with the QD layer. The morphology of the as-fabricated LIG electrodes in the channel region was examined by scanning electron microscopy (SEM). The SEM images reveal the formation of a well-defined channel between the electrodes (Fig. 1c, top), where the graphene morphology exhibits an interconnected filamentary network (Fig. 1c, bottom). This filamentary structure naturally forms during the laser-writing process owing to directional thermal decomposition and carbonization, enabling the filaments to span the electrode gap. Such a continuous and interconnected morphology is expected to facilitate charge transport and enhance the photodetector performance by increasing the contact area with the PbS QDs. Raman spectroscopy was performed to further verify the graphitic nature of LIG, as shown in Fig. 1d. The presence of predominant D (1350 cm^−1^), G (1580 cm^−1^), and 2D (2690 cm^−1^) peaks in the Raman spectrum confirms the formation of a graphene-like structure.^24–27^

**Fig. 2:**
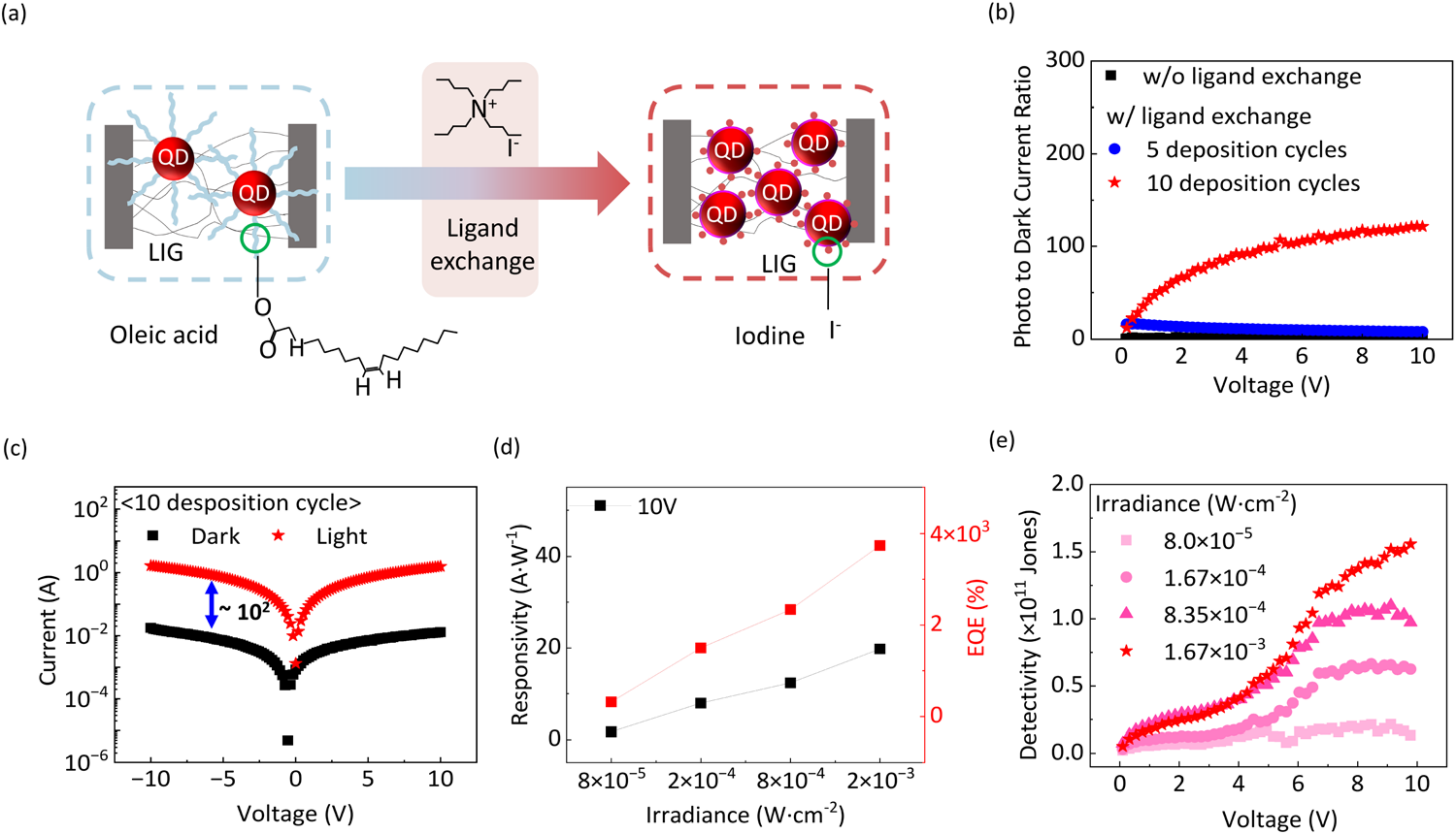
(a) Schematic of QD-LIG heterostructure photodetectors before and after ligand exchange. (b) Photo-to-dark-current ratio with respect to the applied bias of the photodetectors with different QD deposition cycles with ligand exchange. (c) Current–voltage characteristics under dark and illuminated conditions. (d) Responsivity and external quantum efficiency (EQE) of the photodetectors measured at a bias of 10 V with respect to irradiance. (e) Detectivity of the photodetectors measured at different irradiance.

As discussed previously, we conducted solid-state ligand exchange on PbS QDs using TBAI to enhance electronic coupling with LIG. As shown in Fig. 2a, TBAI treatment, followed by rinsing with methanol, effectively replaces the native long-chain oleic acid ligands with shorter iodine-based ligands.^28^ This ligand exchange reduces the inter-QD distance, enabling denser QD packing and facilitating more efficient charge transfer from the QDs to the LIG. In addition to shortening the tunnelling distance, the ligand exchange also modifies the surface potential of the QDs, shifting the conduction-band edge to a deeper energy level relative to the vacuum level (Fig. S4). This shift aligns the QD conduction band more closely with the Fermi level of LIG, which suppresses carrier trapping at interface states. Consequently, the improved band alignment, together with the shorter ligand length, synergistically enhances electron transfer efficiency from the QD layer to the LIG electrode. Upon illumination, the PbS QDs absorb photons and generate electron–hole pairs. These photo-generated charge carriers are separated at the QD-LIG interface and transported towards their respective electrodes under an applied bias, resulting in measurable photocurrent. To optimize the device performance, we varied the thickness of the QD active layer by repeating the deposition cycles, including QD spin-coating, ligand exchange, and methanol rinsing, either five or ten times. Fig. 2b presents the photo-to-dark current ratio (PDCR), a key figure of merit that quantifies the sensitivity of the photodetector, defined as PDCR = *I*_photo_/*I*_dark_ (*I*_photo_ is the current measured when the device is illuminated and *I*_dark_ is the dark current measured without illumination).^28^ Higher PDCR values correspond to improved photosensitivity and signal-to-noise characteristics. Devices fabricated without ligand exchange using native oleic acid-capped QDs exhibit negligible PDCR across the entire measurement voltage range up to 10 V. This poor performance is attributed to the long insulating ligands, which limit the charge transport between the QDs and LIG electrodes. In contrast, devices incorporating iodide ligand exchange show a significant increase in PDCR. Moreover, devices fabricated with the ten coating cycles with ligand exchange demonstrate an enhanced PDCR compared with those fabricated with only five cycles. As confirmed by energy-dispersive X-ray spectroscopy (EDS) elemental mapping (Fig. S5), the QDs are more uniformly and densely distributed across the LIG network in the device with ten coating cycles compared to that with five cycles, supporting the improved interfacial coupling and charge-transfer efficiency. This enhanced uniformity, together with the increased active-layer thickness, contributes to a higher number of photogenerated carriers and superior photodetector performance.^29,30^ Notably, devices with ten coating cycles achieve PDCR values exceeding 100 at a bias voltage of 5 V, indicating excellent photodetection performance.

Fig. 2c shows the current–voltage characteristics of the QD-LIG heterostructure detectors fabricated with ten cycles of QD deposition with ligand exchange, measured under both dark and illuminated conditions. Under illumination, the photocurrent increases by approximately two orders of magnitude compared with the dark current, confirming the strong light responsiveness and efficient photocarrier generation of the device. We further evaluated the spectral responsivity (*R*) of the devices, defined as the ratio of net photocurrent (*I*_photo_ − *I*_dark_) to the incident optical power (*P*_light_), *i.e.*, *R* = (*I*_photo_ − *I*_dark_)/*P*_light_.^31^ As shown in Fig. 2d, the device exhibits spectral responsivity of 1.69, 7.94, 12.4, and 19.8 A W^−1^ at a wavelength of 656 nm under irradiation powers of 8.0 × 10^−5^, 1.67 × 10^−4^, 8.35 × 10^−4^, and 1.67 × 10^−3^ W cm^−2^, respectively. The linear increase in responsivity with increasing light intensity indicates a stable and consistent photoresponse, demonstrating the capability of the device to operate reliably across a broad range of illumination conditions. The responsivity is determined by the amount of photocurrent, which depends on two key factors: (1) the generation rate of charge carriers, which is directly proportional to the intensity of the incident light and optical absorption efficiency of the QDs, and (2) the efficiency of charge transfer from the QDs to the LIG electrodes. The latter is strongly influenced by the interfacial distance between the QDs and graphene. In this study, solid-state ligand exchange using short iodide ligands effectively minimize this distance, thereby improving the charge-transfer rate. The observed linear relationship between responsivity and incident optical power, without any saturation within the measured range, confirms that efficient charge extraction is achieved from the QDs to graphene, enabled by the use of short, conductive ligands. From the measured responsivity, the external quantum efficiency (EQE) can be calculated using the following equation:1
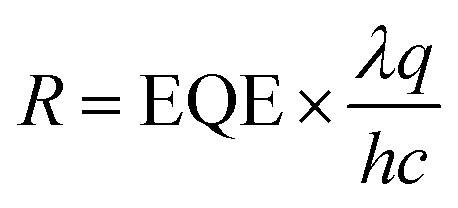
where *λ* is the wavelength of incident light, *q* is the elementary charge, *h* is the Plank's constant, and *c* is the speed of light. The calculated EQE values at various optical powers are shown in Fig. 2d, with the maximum EQE of 3740% at 10 V. Photoconductors typically exhibit photoconductive gain, where increased charge injection under illumination leads to amplified photocurrent and EQE values exceeding 100%. This enhancement is attributed to the modulation of charge injection from the LIG electrodes into the QDs under illumination, leading to an amplified photocurrent. The specific detectivity (D*) was evaluated using the following expression:^31^2
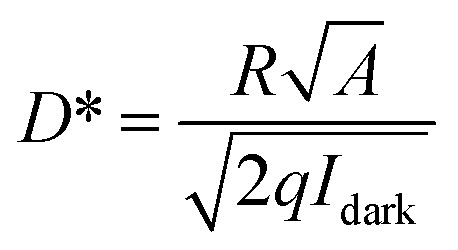
where *A* denotes the effective device area. The bias-dependent specific detectivity is shown in Fig. 2e. An increase in both the applied bias and illumination intensity enhances the specific detectivity. Notably, under an irradiance of 1.67 × 10^−3^ W cm^−2^, a high detectivity exceeding 10^11^ Jones is achieved at 6.5 V, demonstrating the excellent performance of the as-fabricated LIG-QD heterostructure photodetector.

**Fig. 3:**
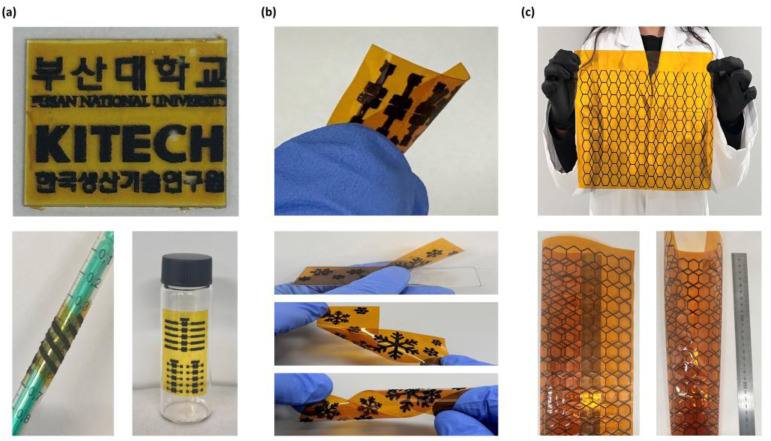
(a) LIG patterns formed directly on flexible PI tape (top, 2 cm × 2.4 cm) that are easily attachable to curvy surface such as syringes (left bottom) and vials (right bottom). (b) Various LIG patterns formed on commercially available flexible PI films featuring its applications in flexible electronics. (c) LIG patterns fabricated on large-area PI substrate (20 cm × 30 cm).

In addition to demonstrated application of LIG in photodetectors, it offers significant advantages for fabricating electrodes that are not only simple and cost-effective, but also compatible with flexible substrates. Fig. 3 shows various examples of LIG patterning on different substrate configurations, including the flexible formats. LIG fabrication is highly adaptable, accommodating multiple forms of PI substrates, such as films, solutions, and tapes, depending on the specific requirements of the application. For instance, Fig. 3a shows that LIG can be directly patterned onto a PI tape, which can then be transferred or attached to arbitrary surfaces, enabling flexible and application-specific integration. LIG-patterned PI tapes can readily adhere to non-planar or curved surfaces, demonstrating excellent mechanical flexibility and substrate conformity for practical deployment. Similarly, as shown in Fig. 3b, PI films enable the formation of LIG electrodes on bendable and conformable platforms, making them suitable for mechanically adaptive electronics. The formed LIG patterns does not change even when the film is bent or deformed. The integrity of the LIG pattern is maintained under mechanical deformation, such as the bending or flexing of the substrate, indicating excellent mechanical robustness. Another advantage of the LIG technology is its compatibility with large-area processing. As shown in Fig. 3c, various LIG patterns are rapidly fabricated over a large area (20 cm × 30 cm), demonstrating the scalability of this technology. These results collectively highlight the versatility of LIG for the fabrication of flexible large-area photodetectors across various form factors. The ability to tailor the electrode design through direct laser patterning on diverse substrates positions LIG as a promising platform for next-generation flexible and scalable optoelectronic devices.

## 4. Conclusions

We demonstrated a heterostructure photodetector platform based on LIG integrated with PbS QDs. The direct laser writing of LIG on PI substrates enabled low-cost, scalable, and lithography-free fabrication of conductive electrode patterns, while its intrinsic porous morphology provided enlarged interfacial contact for efficient charge transfer. Through ligand engineering of PbS QDs and optimization of the QD active layer thickness, the resulting devices exhibited high photosensitivity, an enhanced photo-to-dark current ratio, and strong responsivity, confirming the feasibility of LIG-based electrodes for optoelectronic applications. Specifically, the optimized LIG-QD heterostructure device achieved a photo-to-dark current ratio exceeding 100 at 5 V, a responsivity of approximately 19.8 A W^−1^ at 656 nm under 1.67 × 10^−3^ W cm^−2^ illumination, and a specific detectivity greater than 10^11^ Jones.

Furthermore, the versatility of LIG was demonstrated through its compatibility with various PI formats, including tapes and films, and its ability to form customizable large-area patterns.

Overall, this approach offers clear advantages in terms of fabrication simplicity, cost-effectiveness, and substrate compatibility, establishing LIG as a viable alternative to CVD-grown graphene for large-area and potentially flexible devices. However, limitations remain, including relatively high dark current compared to state-of-the-art photodetectors. Future studies should therefore focus on reducing noise and dark current through refined interfacial engineering, broadening the detection range, and validating device reliability under environmental and mechanical stress. Addressing these aspects will further strengthen the case for LIG-QD heterostructures as a practical platform for next-generation, high-performance, and low-cost optoelectronic systems.

## Author contributions

Jiyoon Oh: writing – original draft, conceptualization, data curation, investigation. Donggu Lee: validation. Kyoungeun Lee: investigation. Yeyun Bae: investigation. Jaeyeop Lee: validation. Yeong Uk Kim: resources. Byeong Guk Jeong: resources. Jaehoon Kim: investigation, Hanchul Cho: conceptualization, project administration, supervision. Jeongkyun Roh: writing – review & editing, supervision.

## Conflicts of interest

There are no conflicts to declare.

## Supplementary Material

RA-015-D5RA07157G-s001

## Data Availability

The data that support the findings of this study are available from the corresponding author upon reasonable request. Supplementary information (SI): additional characterization data, including: sheet resistance characteristics of LIG under various laser-processing conditions, SEM images of LIG with different processing conditions, quantitative analysis of LIG porosity *versus* scan speed and laser power, energy band diagrams of the QD–LIG interface before and after iodide ligand exchange, and EDS elemental mapping of PbS QDs deposited on LIG electrodes. See DOI: https://doi.org/10.1039/d5ra07157g.
