# Supplementary material for: Laser-induced graphene as a versatile platform for colloidal quantum dot heterostructure photodetectors
Source: RSC Adv. 2025 Dec 2;15(55):47360–5. doi: 10.1039/d5ra07157g (PMC12670238; doi:10.1039/d5ra07157g)
Supplement: RA-015-D5RA07157G-s001 [file RA-015-D5RA07157G-s001.pdf]

Supplementary Information (SI) for RSC Advances.
This journal is © The Royal Society of Chemistry 2025

Supporting Information

Laser-Induced Graphene as a Versatile Platform for Colloidal Quantum Dot Heterostructure Photodetectors

Jiyoon Oh[†, a,b], Donggu Lee [c], Kyoungeun Lee [a], Yeyun Bae [a], Jaeyeop Lee [a], Yeong Uk Kim [d], Byeong Guk Jeong [d], Jaehoon Kim [e], Hanchul Cho [b], Jeongkyun Roh [a*]*

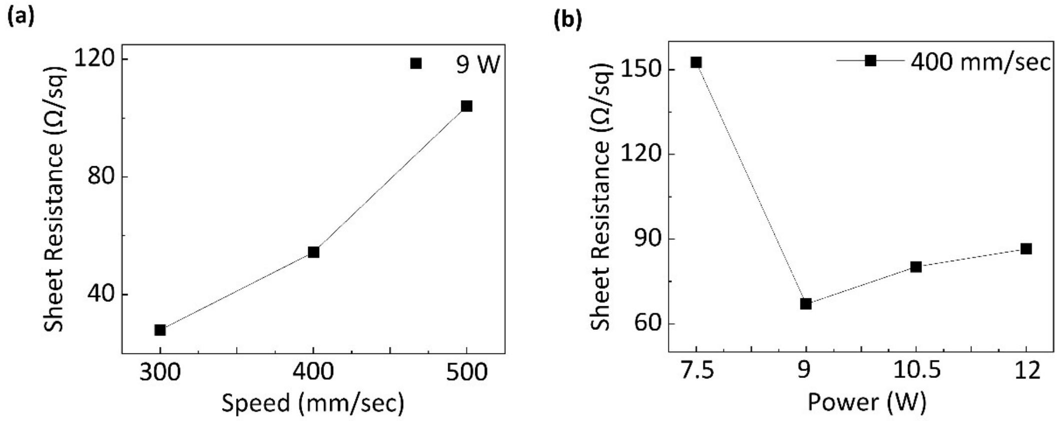

Figure S1. Sheet resistance characteristics of LIG with (a) different scan speed and (b) laser power

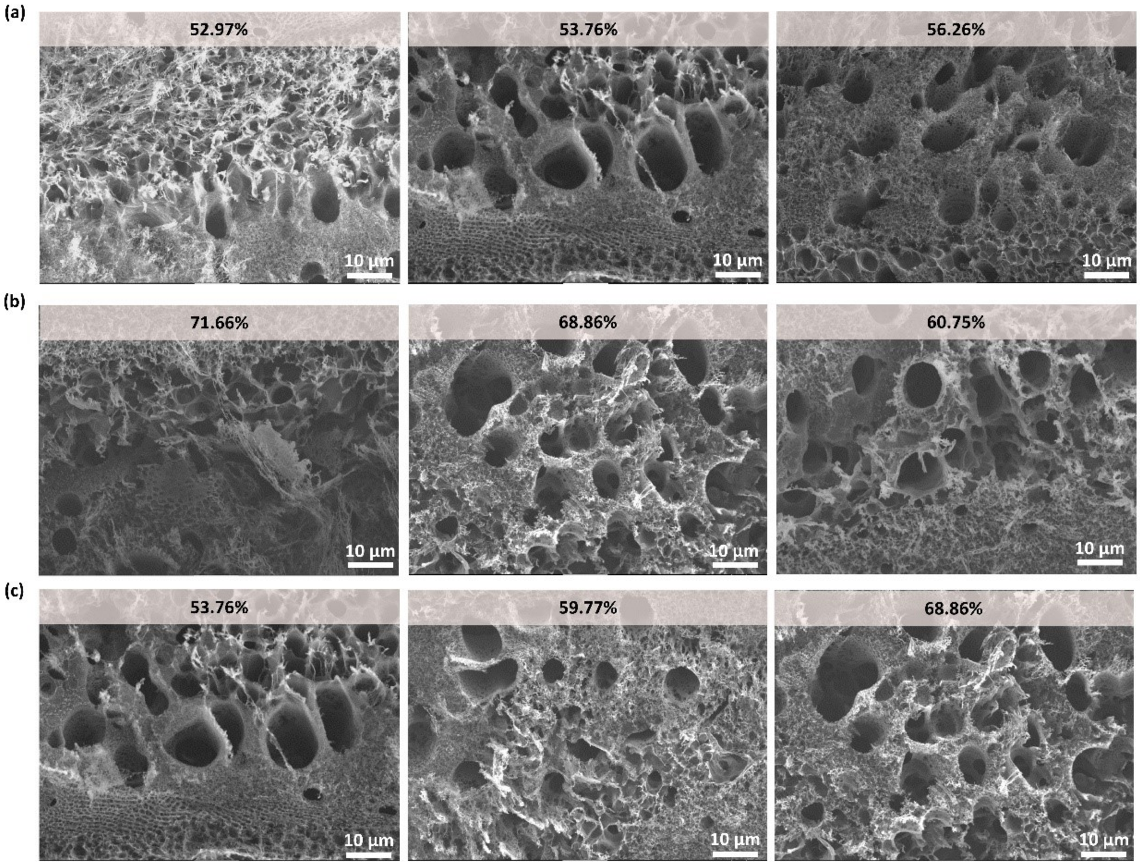

Figure S2. SEM images of LIG with different processing condition: (a) laser power of 9 W at scan speeds of 300, 400, and 500 mm/s (b) laser power of 12 W at scan speeds of 300, 400, and 500 mm/s and (c) fixed scan speed of 400 mm/s with the laser power of 9, 10.5, and 12 W.

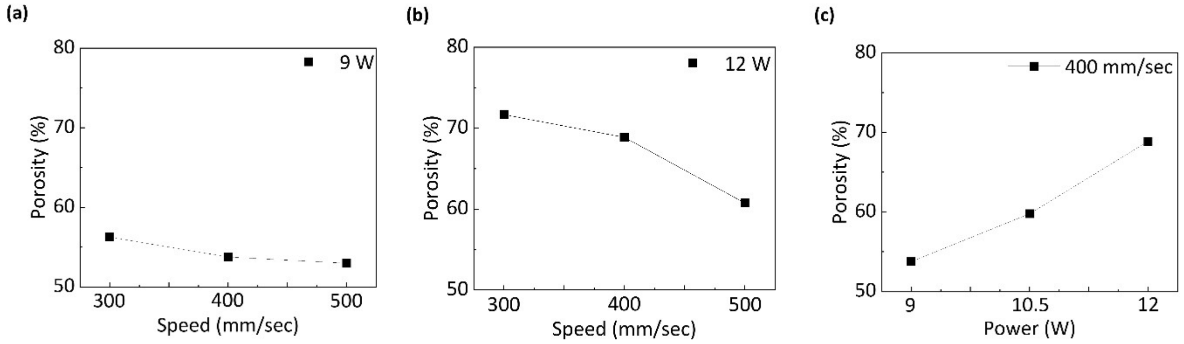

Figure S3. Quantitative analysis of LIG porosity under different laser-processing conditions: (a) porosity variation with increasing scan speed at 9 W, (b) porosity variation with increasing scan speed at 12 W, and (c) porosity change with increasing laser power at a fixed scan speed of 400 mm/s.

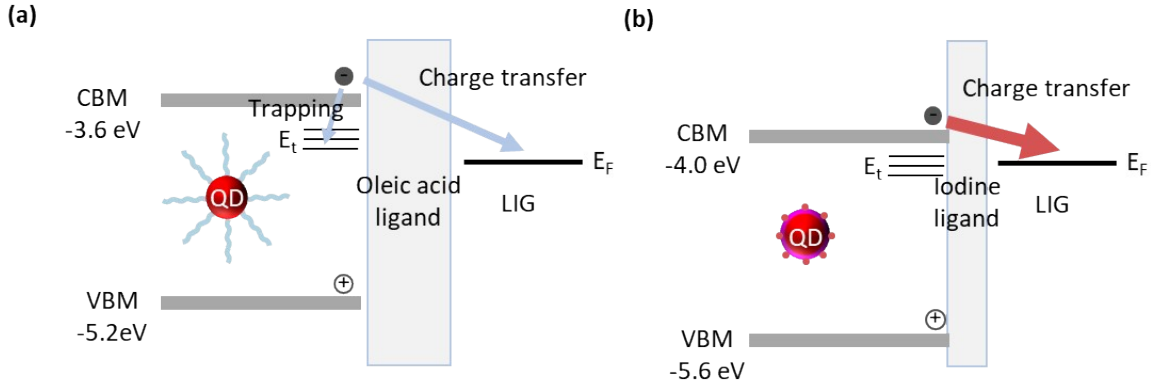

Figure S4. Energy band diagrams of the QD–LIG interface: (a) before and (b) after the iodide ligand exchange process. The ligand exchange shifts the QD conduction band closer to the Fermi level of LIG, resulting in improved band alignment and enhanced charge transfer efficiency across the interface.

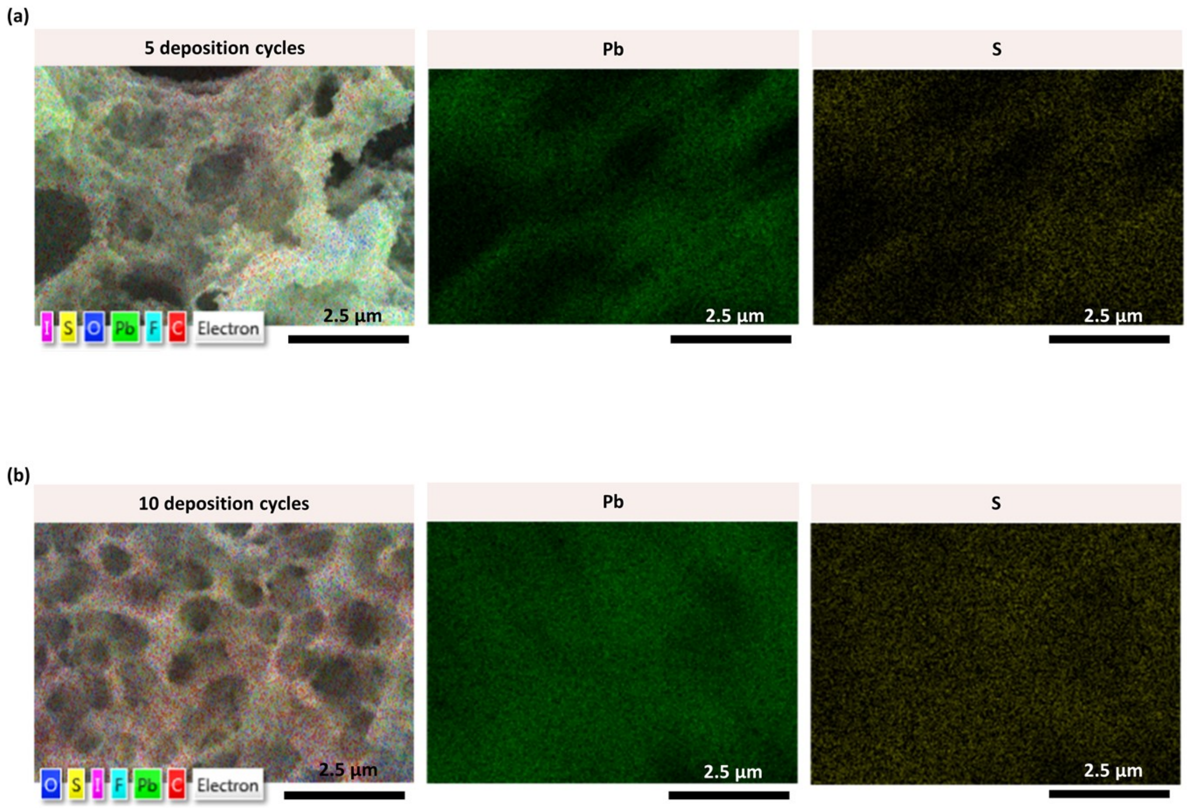

Figure S5. Energy-dispersive X-ray spectroscopy (EDS) elemental mapping of PbS QDs for the samples with (a) five and (b) ten ligand-exchange cycles.
